# Evaluating Health Literacy in Virtual Environments: Validation of the REALM and REALM-Teen for Virtual Use

**DOI:** 10.1007/s11606-022-07474-9

**Published:** 2022-03-21

**Authors:** Julie L Aker, Terry C Davis, Andrea Leonard-Segal, Lori Christman, Sara Travis, Melissa Beck, Angela Newton

**Affiliations:** 1 Concentrics Research, 9335 Delegates Row, Indianapolis, IN 46240 USA; 2 Louisiana State University Health Shreveport, Shreveport, USA; 3 George Washington University School of Medicine and Health Sciences, Washington, DC USA; 4 Concentrics Research-STATKING JV, Indianapolis, USA

## INTRODUCTION

It is important to include populations of diverse literacy levels in healthcare research. The coronavirus disease 2019 (COVID-19) pandemic highlighted the need for virtual approaches to evaluate health literacy for research studies including those focused on drug development for potential FDA approval. We were unable to find evidence of a validated methodology to assess health literacy during video visits or on mobile devices.

Health literacy, defined as the degree to which individuals have the capacity to obtain, process, and understand basic health information and services needed to make appropriate health decisions,^1–3^ is known to affect patient understanding and outcomes.^4–9^ There is an ongoing need for health literacy to be considered in the development of health information, prescription and over-the-counter (OTC) drug labeling, and patient prescription medication guides.^10–13^ To provide mechanisms for continuity of research programs during the pandemic, the US Food and Drug Administration published a Guidance for Industry^14^ to encourage the use of virtual study visits.

Two of the most commonly used instruments to evaluate health literacy in adult and adolescent research are The Rapid Estimate of Adult Literacy in Medicine (REALM)^15,16^ and the Rapid Estimate of Adolescent Literacy in Medicine (REALM-Teen).^17^ However, these have only been validated for face-to-face administration.

The purpose of this study was to validate the use of the REALM and REALM-Teen for virtual use by video interview. We hypothesized that using the participant’s own mobile device or computer would provide a fast and convenient method to evaluate health literacy.

## METHODS

Concentrics Research identified studies they had recently conducted that also included an in-person REALM test. Twelve research sites across the USA that partnered with Concentrics on these studies contacted the study participants. To avoid selection bias, a random number generator created a random sampling schema used to identify potential participants. These potential participants were stratified by their previous REALM or REALM-Teen scoring categories based on the REALM scoring interpretation (Table 1). In accordance with the FDA Code of Federal Regulations, this virtual REALM test did not require an IRB review since it is not regarded as a clinical investigation.^18^

**Table 1:** Interpretation Scores for REALM and REALM-Teen

REALM interpretation scores^*^	
**Raw score**	**Grade range equivalent**
0–18	3^rd^ grade and below
19–44	4th to 6th grade
45–60	7th to 8th grade
61–66	9th grade and above
**REALM-Teen score interpretation**	
**Raw score**	**Grade range equivalent**^†^
0–37	3^rd^ grade and below
38–47	4^th^ to 5^th^ grade
48–58	6^th^ to 7^th^ grade
59–62	8^th^ to 9^th^ grade
63–66	10^th^ grade and above

^*^Source: Davis TC, Long SW, Jackson RH, et al. Rapid estimate of adult literacy in medicine: a shortened screening instrument. Fam Med. 1993 Jun;25(6):391-5. PMID: 8349060

^†^Source: Davis TC, Wolf MS, Arnold CL, et al. Development and validation of the Rapid Estimate of Adolescent Literacy in Medicine (REALM-Teen): a tool to screen adolescents for below-grade reading in health care settings. Pediatrics. 2006; 118:e1707-14

### Participants

Trained recruiters contacted adults ages 19 to 77 and adolescents ages 12 to 19 who had participated in a previous study with a face-to-face administration of the REALM or REALM-Teen to invite them to participate in the study. Recruiters advised potential participants that the interview would be conducted using their personal device (e.g., an Android or iOS smartphone, a tablet, or a computer). Individuals interested in participating were scheduled for the virtual visit.

Eligibility criteria (inclusion and exclusion criteria) were confirmed (Table 2). These criteria are consistent with best practices and historic expectations from FDA for consumer research studies in OTC drug development programs^19^ (e.g., label comprehension, human factors, self-selection, and actual use).

**Table 2:** Criteria for Inclusion and Exclusion

**Criteria for inclusion:** **Population (*n*= 200 REALM and*n*= 200 REALM-Teen)** 1. Are male or female, of any race 2. 12 years (6^th^ grade) of age or older 3. Able to read, speak, and understand English 4. Access to internet and have a smartphone, tablet, or computer 5. Read and verbally consent to the Agreement to Participate (if 18 years of age or older) or Assent (if under 18 years of age) 6. Adolescents only: parent of adolescent participant under the age of 18 provides permission 7. Have a previous in-person REALM/REALM-Teen score 8. Adolescents only: if previous score is from REALM-Teen, then must meet the age requirements for the REALM-Teen	
**Criteria for exclusion:** Participants will be excluded from the study if: 1. The participant or anyone in his/her household is currently employed by any of the following: a. A marketing, marketing consulting, or marketing research company b. An advertising agency or public relations firm c. A pharmacy or pharmaceutical company d. A manufacturer of medicines e. A managed care or health insurance company as a healthcare professional f. A healthcare practice 2. The participant has ever been trained or employed as a healthcare professional. 3. The participant normally wears corrective lenses, contacts, or glasses to read and does not have them. 4. The participant has any other impairment that prevents him/her from being able to read on his/her own. 5. The participant is not willing or able to comply with the study procedures.	

The 19-month past study window was selected to increase the likelihood of successfully contacting study participants and to increase the probability that adolescents would be as close as possible to the grade level they were when they completed the in-person REALM-Teen test. The moderators were trained and experienced healthcare researchers located centrally at Concentrics Research in Indianapolis, Indiana.

### Study Instruments

The instruments used in this testing were word recognition tests which are useful predictors of reading ability in English. If an individual has difficulty pronouncing words in isolation, which is a beginning-level reading skill, he or she is likely to have difficulty with comprehension (a higher-level skill). An individual’s reading grade level is commonly used as an indicator of his/her health literacy.

#### REALM

Adult health literacy was assessed using the REALM, a widely used, validated screening test consisting of 66 commonly used lay medical terms arranged in order of increasing difficulty.^15,16^ Raw REALM scores (0–66) can be converted into reading grade levels. A score < 61 indicates a reading level of 7^th^–8^th^ grade or below, an indicator of limited literacy. A score ≥ 61 indicates a reading level of 9^th^ grade and above, an indicator of adequate literacy (Table 1).

#### REALM-Teen Test

The REALM-Teen is a validated word recognition screening test consisting of 66 adolescent-appropriate health words arranged in order of increasing difficulty.^17^ All words were chosen from the American Academy of Pediatrics’ adolescent patient education materials. The test provides an estimate of each subject’s reading grade range and detects below-grade reading level (Table 1).

#### Virtual REALM/REALM-Teen Tests

**Figure 1:**
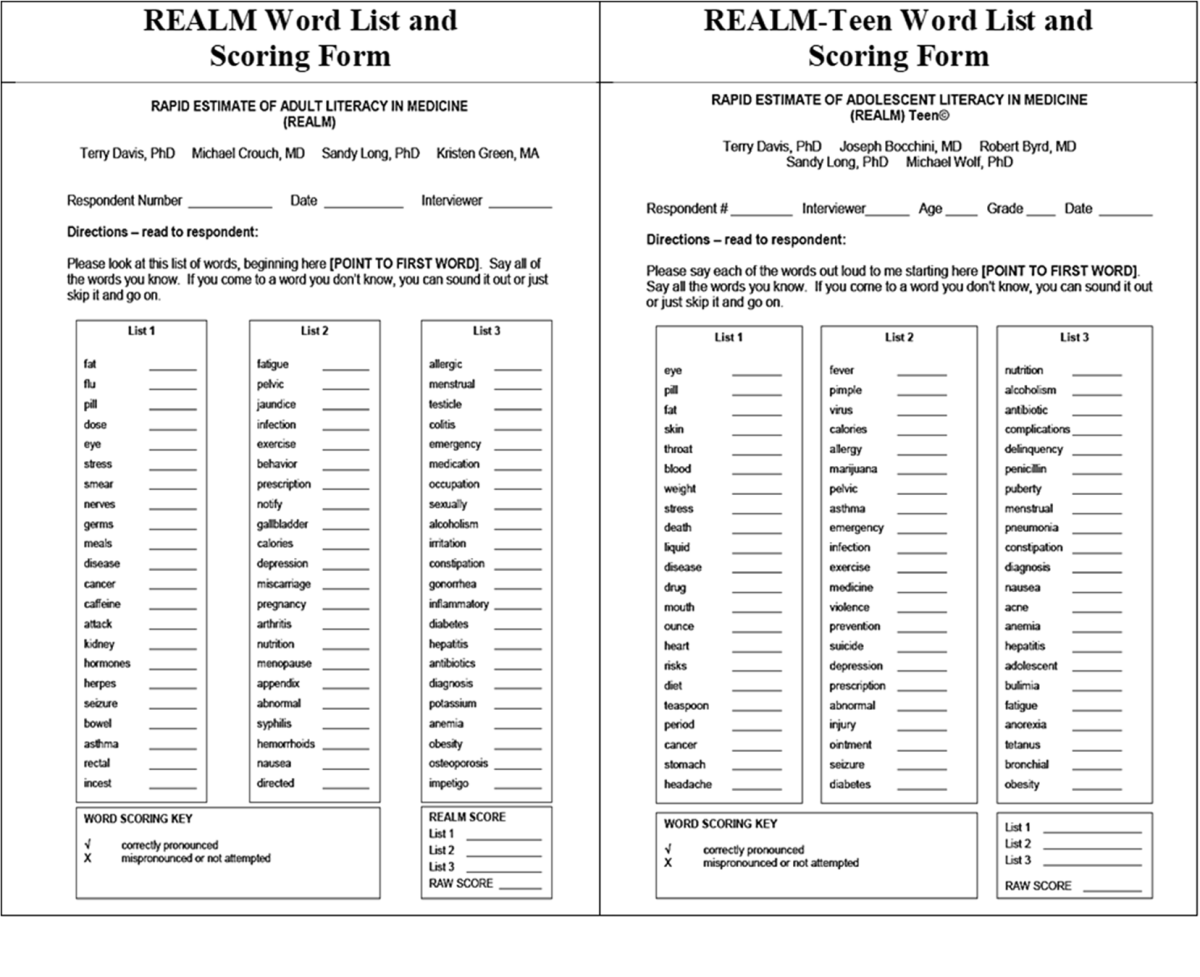
REALM word lists and scoring forms.

**Figure 2:**
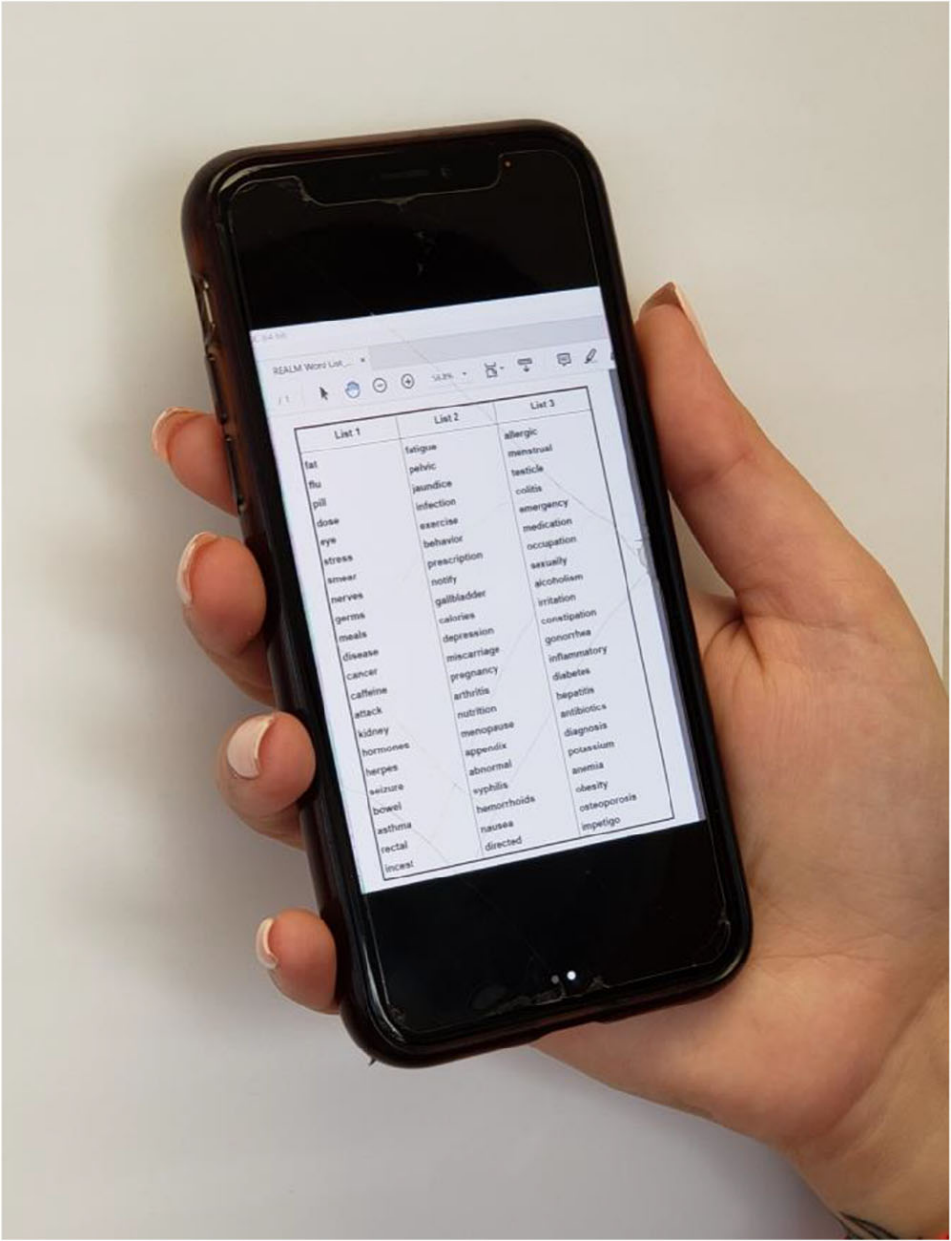
REALM word list on a smartphone.

The virtual REALM and virtual REALM-Teen instruments were comprised of the respective REALM/REALM-Teen Word List (Fig. 1) displayed on the moderator’s computer during the video visit and viewed on the participant’s device of choice (Fig. 2).

#### Post-Test Survey

The purpose of this survey was to gain feedback about participants’ experience with the virtual REALM test format. The survey also gathered information about how study participants had used technology devices to access healthcare information (e.g., shopping for medicines online, searching for information) and how they might want to use technology in the future (e.g., electronic labeling, easy-to-access information about drugs, drug interactions, side effects, dosing, warnings, and directions, as well as leaflets and/or education).

A 6-question post-test survey administered by the moderators included the following questions: 1. Did you have any difficulties or suggestions related to this video interview?

2. What types of healthcare products, if any, do you purchase on your own?

3. How do you currently use your technology device to get healthcare information?

4. In the future, what medication information would you like to be able to access electronically?

5. In the future, what *type of information* about drugs would be most important for you to get electronically?

6. What would be the *easiest way for you to access information* about medications?

### Study Design

Prior to the interview, the study staff conducted a technology check with the participant to ensure that he/she had internet access, to confirm the device being used, and to ensure that he/she knew how to use the audio and video controls for the videoconference. This was a single-visit virtual interview study; all interviews were recorded. Privacy was protected through the use of participant identification numbers. Databases were all on secure servers.

Once the video interview was initiated, the participant was welcomed and provided with a brief study overview. Adult participants were provided with a consent form, and adolescents were provided with an assent form they could view on their device screen. These forms included the study purpose, the study activities, benefits, risks, and statement that the research participation was voluntary. Interested adults (18 years of age and older) provided their consent. Adolescents (12–17 years of age) were provided with an assent form to obtain their agreement to participate, and their parent was required to provide permission for the interview.

The moderator provided the REALM word list to adult participants or the REALM-Teen word list to adolescent participants (Fig. 1) to view on their device (Fig. 2) during the testing. As with the in-person REALM/REALM-Teen, each test was administered by providing the word list to the participant and asking him/her to read the words on the list aloud. The moderator scored which words were pronounced correctly or incorrectly. The correctly pronounced words corresponded to the REALM scores (Table 1). The REALM and REALM-Teen tests are validated to be administered face-to-face; therefore, video interviews were conducted so that the moderator and participant could see each other throughout the interview, just as they would during an in-person assessment. Following the virtual test, the moderators conducted the post-test survey.

### Statistical Analysis

Demographic variables and baseline participant characteristics for the study included age, REALM/REALM-Teen score from in-person evaluation, time from in-person REALM/REALM-Teen to enrollment into this study, sex, race, ethnicity, REALM / REALM-Teen score category, education, income, and employment. Continuous variables were summarized with descriptive statistics (mean, standard deviation (SD), number of nonmissing observations, minimum, maximum, and median) within each participant group (adults, adolescents), while categorical variables were summarized using frequency counts and percentages within each participant group. The analysis of demographic and baseline data was based on the per-protocol (PP) population, which was defined as all participants enrolled in the study who completed the REALM-V or REALM-Teen-V assessment.

The primary endpoint of the study was Pearson’s correlation coefficient between the numerical scores of the in-person REALM (or REALM-Teen) test and the REALM-V (or REALM-Teen-V) test. The sample size was based on powering the study for the primary endpoint for each participant group (adult, adolescent). Assuming the true correlation for the adult population is 0.8, a total sample size of 199 participants in each group would have 90% power to demonstrate that the true correlation is greater than 0.7 at a 1-sided 0.025 significance level.

To enable a meaningful sample size estimation, a preliminary analysis of data from adult and adolescent participants who took the REALM or REALM-Teen test in consecutive years from 2018 to 2020 showed an estimated correlation of 0.9454 using a total of 45 paired data points for adults and 0.9177 using a total of 38 paired data points for adolescents (i.e., within-group pairs between 2018 and 2019, and within-group pairs between 2019 and 2020).

Note that in this preliminary assessment, some participants in both the adult and adolescent groups contributed 2 pairs of observations if they had data from all 3 years. A conservative estimate of 0.8 for the correlation was used for these sample size calculations. Thus, the total sample size planned for each group was rounded up to 200 participants, for a total of 400 participants in the study. Analyses were performed separately for adults and adolescents. All data analyses were performed on the observed data pooled across all research sites. The estimate and 2-sided 95% CI for Pearson’s correlation coefficient, *ρ*, was computed for each participant group. The following hypotheses were tested at a 1-sided *α* = 0.025 level of significance:

H0: *ρ* = 0.7 vs. H0: *ρ* > 0.7.

The null hypothesis was rejected in favor of the alternative if the lower limit of the 2-sided 95% CI for *ρ* was greater than 0.7. The success threshold for correlation was set to 0.7, as values between 0.7 and 1.0 indicate a strong positive linear relationship.^20^ The analyses of the primary endpoints were based on the PP population. All data analyses used SAS version 9.4 (SAS Institute Inc., Cary, NC, USA).

Responses to the post-test survey were coded into categories and summarized with descriptive statistics.

## RESULTS

Overall, 381 participants completed the study (adults = 202; adolescents = 179). Twelve research sites throughout the USA completed participants (Los Angeles: 76; Chicago: 37; Dallas: 8; Denver: 36; Indianapolis: 136; Charlotte: 4; Raleigh: 27; San Francisco: 6; St. Louis: 21; Tampa: 28; Baltimore: 2), thereby providing a geographically diverse population for the analysis.

The adult population was comprised of females (68.8%) and males (31.2%) with ages ranging from 19 to 77 years (mean = 46 years). Races were comprised of Caucasian (58.4%), African-American (27.7%), and other/multicultural (11.4%); 14.4% of the adults were of Hispanic ethnicity. The REALM scores for these participants ranged from 32 to 66 and the time between the in-person REALM to the REALM-V was an average of 10.4 months (Table 3).

**Table 3:** Demographic Characteristics (PP Population, *N* = 381)

**Characteristic**	**Adults** **(*n*= 202)**	**Adolescents** **(*n*= 179)**
Age (years), *n *(%)		
Mean (SD)	46.02 (16.31)	16.11 (1.89)
Median (min, max)	46 (19, 77)	17 (12, 19)
Sex, *n* (%)		
Female	139 (68.81)	148 (82.22)
Male	63 (31.19)	32 (17.78)
Race, *n* (%)		
Native American	1 (0.50)	0 (0.00)
Asian or Pacific Islander	4 (1.98)	6 (3.33)
African-American/Black	56 (27.72)	49 (27.22)
Caucasian/White	118 (58.42)	103 (57.22)
Other	23 (11.39)	22 (12.22)
Ethnicity, *n* (%)		
Hispanic	29 (14.36)	26 (14.44)
Not Hispanic	173 (85.64)	154 (85.56)
In-person REALM/REALM-Teen, *n *(%)		
Mean (SD)	60.61 (6.98)	60.51 (6.70)
Median (min, max)	64 (32, 66)	63 (23, 66)
Time (months) between enrollment and in-person REALM/REALM-Teen Score, *n *(%)		
Mean (SD)	10.43 (3.43)	12.16 (5.26)
Median (min, max)	12 (5, 16)	13 (3, 19)

The adolescent population was comprised of females (82.2%) and males (17.8%) with ages ranging from 12 to 19 years (mean = 16 years). Races were comprised of Caucasian (57.2%), African-American (27.2%), and other/multicultural (12.2%); 14.4% of the adolescents were of Hispanic ethnicity. The REALM-Teen scores for these participants ranged from 23 to 66 and the time between the in-person REALM to the REALM-Teen-V was an average of 12.2 months (Table 3).

For adults, a total of 202 paired observations were included in the analysis. The point estimate of Pearson’s correlation coefficient was 0.918. The lower limit of the 2-sided 95% CI for *ρ* was 0.893, successfully demonstrating a significant correlation that exceeded the 0.7 threshold between REALM-V and in-person REALM tests (Table 4).

**Table 4:** REALM/REALM-V Correlation Analysis (PP Population)

	Number of paired observation	Pearson’s correlation coefficient	95% confidence interval
Adult participants	202	0.918	(0.893, 0.937)
Adolescent participants	179	0.846	(0.797, 0.882)

For adolescents, a total of 179 paired observations were included in the analysis. The point estimate of Pearson’s correlation coefficient was 0.846. The lower limit of the 2-sided 95% CI for *ρ* was 0.797, successfully demonstrating a statistically significant correlation that exceeded the 0.7 threshold between REALM-Teen-V and in-person REALM Teen tests (Table 4).

For the post-test survey, nearly all adult and adolescent participants responded (92.6% [187 participants] and 97.2% [175 participants], respectively) that they had no difficulties or suggestions interacting with an interview conducted by video. Adults and adolescents responded that they primarily use technology to search for information and to access health portals, and they would like to have electronic access to medication information in the future for both prescription and OTC medications, particularly with regard to side effects, dosing, warnings, and directions.

## DISCUSSION

The COVID-19 pandemic created a pressing need for clinicians and researchers to quickly adapt to the use of virtual interactions with patients and research participants. Therefore, we sought to adapt the REALM and REALM-Teen to a virtual environment. We demonstrated a high correlation between the in-person and virtual administration of the REALM and REALM-Teen and provided evidence in a diverse population that supports the use of these well-established instruments in a virtual environment.

In this study, participants demonstrated the ability to easily view the REALM and the REALM-Teen word lists on their devices. If using a smartphone, they intuitively scrolled or pinched the screen to increase or decrease the size, if needed. We observed that very few needed to change the size or orientation of the REALM list on their device (Fig. 2); the word lists fit nicely on one screen even with a smaller smartphone screen. We routinely observe similar behavior during in-person interviews when participants move a REALM word list closer to their face; in fact, some older adults carry a magnifying glass with them routinely for easier reading and we permit this in research studies.

In the post-test survey, study participants expressed that they had no difficulty interacting with the virtual REALM/REALM-Teen test on their device. This is not surprising because, in our research experience, we have observed that study participants appreciate using their own devices during virtual interactions since this eliminates confusion with unfamiliar devices. We have also observed that study participants behave similarly in virtual and in-person interactions, talking freely to the moderator that they can see live on their video screen.

It is important to mention that we chose to conduct video versus phone interviews since the REALM was originally validated for face-to-face administration. Video interviews allow the researcher to confirm that the study participant is reading the word list and, importantly, is not receiving help from anyone else. It also allows the researcher to observe pauses which may indicate difficulty with one or more words versus being distracted by something in the home environment.

Virtual research studies provide for safe and convenient interactions between study participants and researchers. While the general public uses a variety of technology devices, smartphones are the most ubiquitous. The share of Americans who own smartphones was 85% in 2020, up from just 35% in 2011.^21^ Since mobile devices are accessible to a broad population, including those with lower incomes, education, and literacy,^22^ this new virtual method provides a convenient and effective way to evaluate health literacy.

### Limitations

We note three study limitations. Firstly, future studies may be useful to include other languages. This study validated the virtual REALM and REALM-Teen in English. Secondly, the study population is comprised of volunteers who did not have REALM scores at the third grade level or below. Based on over 1000 clinical and consumer studies that we have conducted utilizing REALM testing, it is unusual for people with extremely limited literacy to volunteer for studies that necessitate reading study materials such as an informed consent, study instructions, or medication labeling. Thirdly, there is a higher proportion of females in this study. The population was comprised of volunteers from 53 studies that had no pre-established quotas for sex demographics. It is our experience that it is more common for women to volunteer for clinical and consumer studies than it is for men. Similarly, there were more women than men in the validation studies of the REALM^15^ (82% females), REALM-Teen^17^ (53% female), and New vital sign^23^ (83% female).

## CONCLUSIONS

This study validated the use of the REALM and REALM-Teen in a virtual setting with the use of smartphones, tablets, or computers across the adult and adolescent populations. It demonstrated that the virtual interview offers a practical and reliable approach for conducting the REALM and the REALM-Teen test to assess health literacy.
